# Early Changes of Articular Cartilage and Subchondral Bone in The DMM Mouse Model of Osteoarthritis

**DOI:** 10.1038/s41598-018-21184-5

**Published:** 2018-02-12

**Authors:** Hang Fang, Lisi Huang, Ian Welch, Chris Norley, David W. Holdsworth, Frank Beier, Daozhang Cai

**Affiliations:** 1 Department of Orthopedics, The Third Affiliated Hospital of Southern Medical University, 183 Zhongshan Ave. West, Guangzhou, 510630 P.R. China; 2 Department of Physiology and Pharmacology, Schulich School of Medicine & Dentistry, Western University, 1151 Richmond Street, London, ON N6A 5C1 Canada; 3 Academy of Orthopaedics, Guangdong Province, 183 Zhongshan Ave. West, Guangzhou, 510630 P.R. China; 4 Orthopaedic Hospital of Guangdong Province, 183 Zhongshan Ave. West, Guangzhou, 510630 P.R. China; 5 Department of Clinical Laboratory, Sun Yat-sen Memorial Hospital of Sun Yat-sen University, Guangzhou, 510120 P.R. China; 6 Animal Care and Veterinary Services, Western University, 1151 Richmond Street, London, ON N6A 5C1 Canada; 7 Imaging Research Laboratories, Robarts Research Institute, P.O. Box 5015, 100 Perth Drive, London, ON N6A 5K8 Canada

## Abstract

To examine the early changes of articular cartilage and subchondral bone in the DMM mouse model of osteoarthritis, mice were subjected to DMM or SHAM surgery and sacrificed at 2-, 5- and 10-week post-surgery. Catwalk gait analyses, Micro-Computed Tomography, Toluidine Blue, Picrosirius Red and Tartrate-Resistant Acid Phosphatase (TRAP) staining were used to investigate gait patterns, joint morphology, subchondral bone, cartilage, collagen organization and osteoclasts activity, respectively. Results showed OA progressed over 10-week time-course. Gait disparity occurred only at 10-week post-surgery. Osteophyte formed at 2-week post-surgery. BMDs of DMM showed no statistical differences comparing to SHAM at 2 weeks, but BV/TV is much higher in DMM mice. Increased BMD was clearly found at 5- and 10-week post-surgery in DMM mice. TRAP staining showed increased osteoclast activity at the site of osteophyte formation of DMM joints at 5- and 10-week time points. These results showed that subchondral bone turnover might occurred earlier than 2 weeks in this mouse DMM model. Gait disparity only occurred at later stage of OA in DMM mice. Notably, patella dislocation could occur in some of the DMM mice and cause a different pattern of OA in affected knee.

## Introduction

Osteoarthritis (OA) is a prevalent musculoskeletal disease that causes pain and disability for millions of people. It is characterized by progressive cartilage erosion, subchondral bone turnover and osteophyte formation, amongst other pathological alterations within the joint. Recent findings suggested that subchondral changes might precede cartilage degeneration during OA^1^. Gene expression of subchondral bone in a rat model was reported dramatically dysregulated before noticeable articular cartilage damage^2^. The correlation between bone remodeling and OA was elegantly reviewed by Burr and Gallant^3^. Sanchez *et al*. found subchondral bone osteoblasts can induce phenotypic changes in human osteoarthritic chondrocytes^4^, downregulate aggrecan but upregulate metalloproteinases expression by chondrocytes^5^. Not only subchondral bone can affect cartilage in OA, but also cartilage can affect subchondral bone. Stock *et al*. found UPMA from cartilage can be secreted into the cartilage matrix and may migrate to the cartilage-bone interface, causing subchondral bone changes in the process of OA^6^. All evidence points to a greater role that subchondral bone plays in OA initiation and progression. However, how articular cartilage and subchondral bone changes during the time-course of OA in the predominant model of post-traumatic OA—the DMM model, has not yet been fully described. Understanding the articular cartilage and subchondral bone changes (especially in the early stage) in this commonly used mouse model will provide more information on the pathophysiology of OA.

The DMM (destabilization of the medial meniscus) model is now the most commonly used surgical model in mice. Its reliability, reproducibility, structural similarity to human OA, its relatively slow progression when compared to other surgical models, and the validation of several pain endpoints make it a good model for early OA exploration. Huang *et al*. reported the age-dependent changes in the articular cartilage and subchondral bone in this DMM model and found age and gender matter in articular cartilage and subchondral bone changes after surgery^7^. Patricia Das Neves Borges *et al*. described a comprehensive way to automated segment subchondral bone compartments and assess the bone changes in mice using contralateral knee as controls^8^. However, subchondral bone changes during the time-course of the disease in the DMM model have not been characterized in detail.

This current study aims to examine the early changes of articular cartilage, subchondral bone and their progressions in the DMM model, as well as other parameters of OA including standard histology and gait pattern alterations.

## Results

### Gait disparity

**Figure 1:**
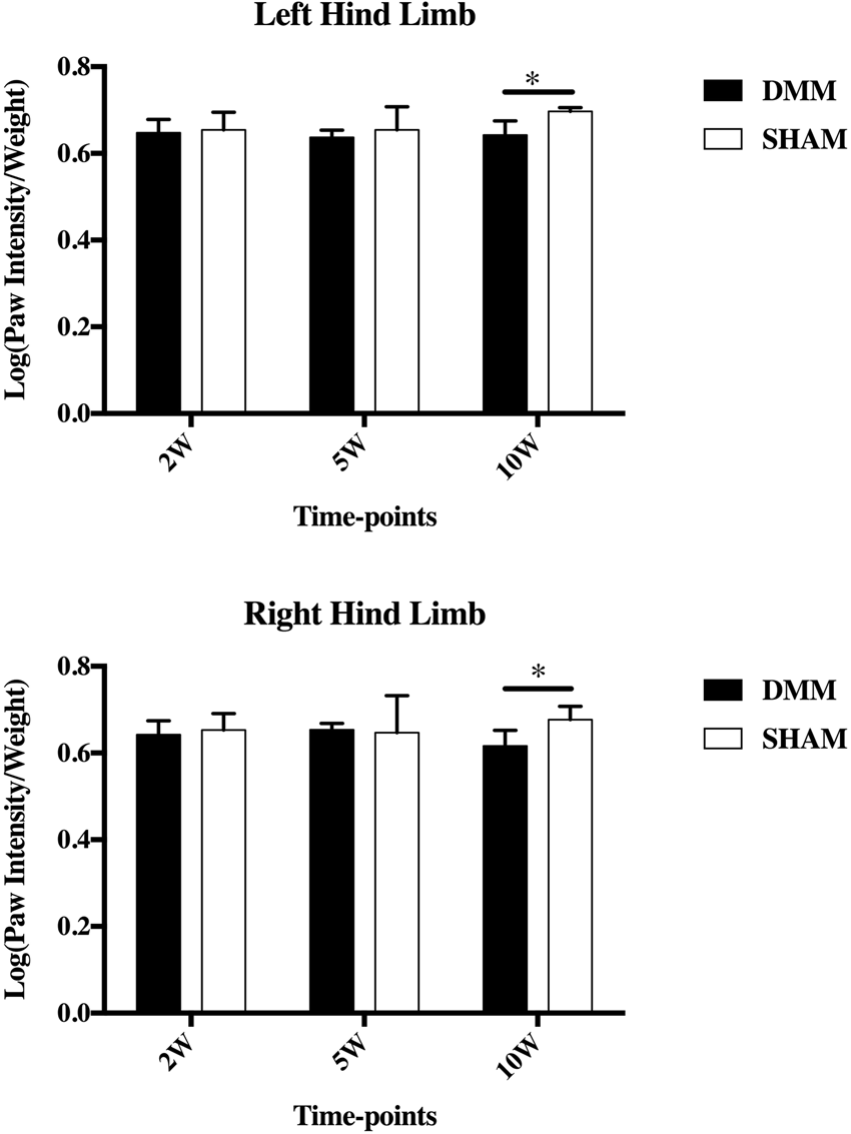
Catwalk gait analysis after DMM surgery. Catwalk analyses demonstrated that paw intensity of both hind limbs was lower in DMM mice compared to SHAM at the 10-week time point. DMM surgery was performed in the left hind limb and the right limb was not operated. No statistical significance was detected at the earlier time-points. Error bar stands for the 95% confidence interval.

Gait disparity only occurred at 10 weeks post-surgery in the DMM group compared to SHAM but not at 2- and 5- week time-points after surgery (Fig. 1). DMM mice at 10-week time-point showed lower paw intensity than SHAM mice in the operated left leg. However, the same effect was observed for the contralateral un-operated leg (Fig. 1).

### Histological analyses of joint changes after DMM surgery

**Figure 2:**
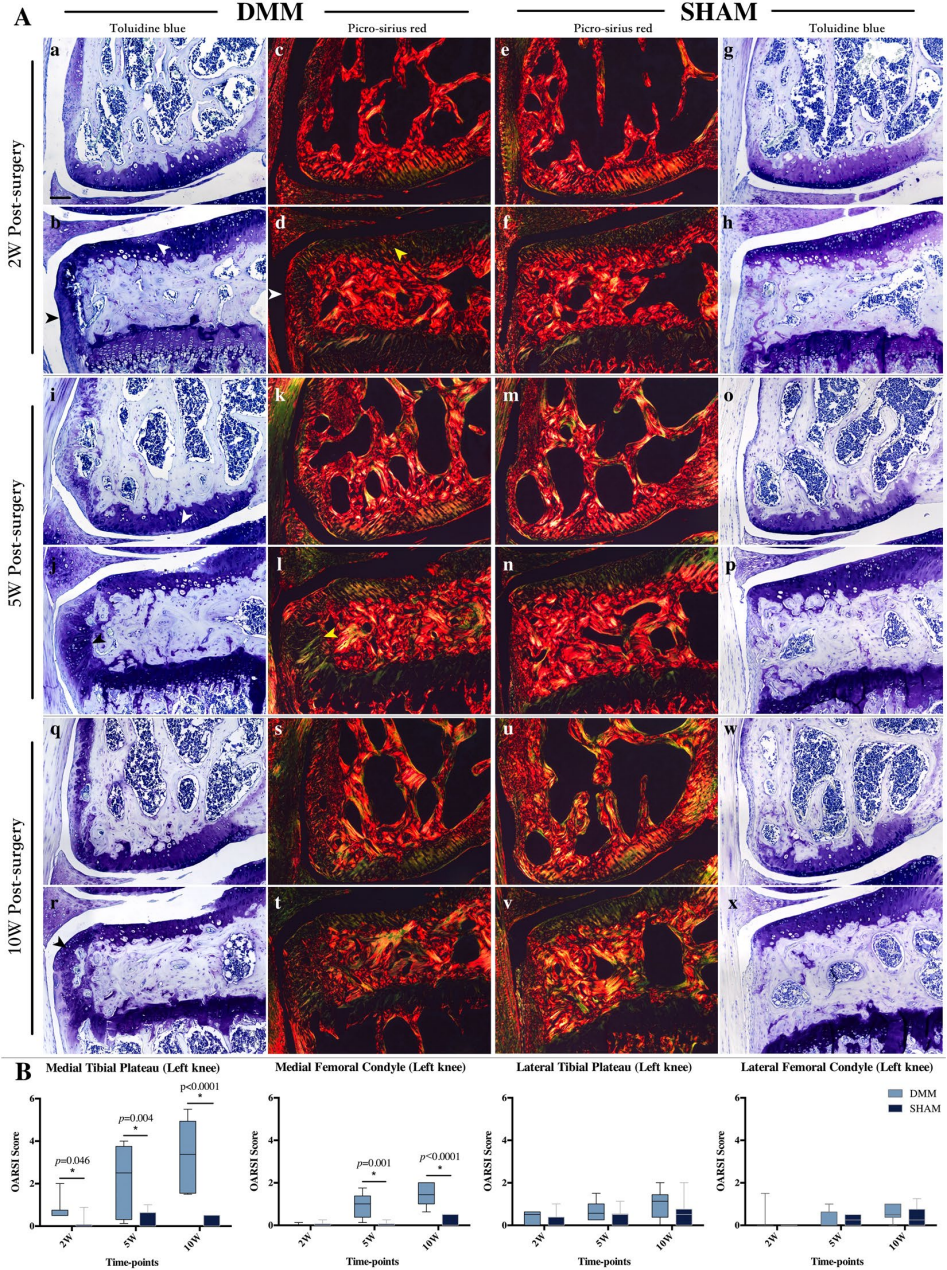
Histological analyses of joint changes after DMM surgery. (**A**) Toluidine blue and picrosirius red staining demonstrated that OA progressed from mild to moderate or severe through the 10-week time course on the medial tibial plateau. Regional proteoglycan loss (b, white arrow), chondrocyte clustering and cartilagenous osteophyte formation (b, black arrow) can be detected at 2 weeks post-surgery. Picrosirius red staining showed changes in osteophyte development from 2- to 10-week post-surgery (d,l,t). Scale bar: 200 µm. (**B**) OARSI scores of the medial tibial plateau of DMM mice were increased at all time-points, while statistically significant increases in the score of the DMM medial femoral condyle was observed at 5- and 10-week time-points (**B**). No statistical difference in scores was seen on the lateral side of the joint.

OA phenotype after DMM surgery was confirmed by histology using toluidine blue (TB) staining. Regional proteoglycan loss and chondrocyte clustering occurred on the medial compartments of the joint at 2 weeks post-surgery, especially the medial tibial plateau (MTP) (Fig. 2A,b, white arrow). Collagen disorganization was also shown in this small region in adjacent sections by picrosirius red (PR) staining (Fig. 2A,d, yellow arrow). Cartilaginous tissue formed on the medial edge of tibia and stained strongly with TB at 2-week time-point in DMM mice (Fig. 2A,b, black arrow), with collagen fibril organization similar to that of articular cartilage (Fig. 2A,d, white arrow). 5 weeks post-surgery, mild to moderate cartilage damage can be found in the surgical joints of DMM mice. Cartilage erosion and fibrillation, as well as large osteophytes were noticed on the MTP. Proteoglycan loss and fibrillation were also present on the medial femoral condyle (MFC) (Fig. 2A,i, white arrow). Cartilage lesion on the MTP was deep into the calcified zone 10 weeks post-surgery(Fig. 2A,r). Endochondral ossification can be observed in the osteophyte with bone marrow formed within the osteophyte (Fig. 2A,r, black arrow).

Osteoarthritis Research Society International (OARSI) histopathology scoring system was applied to evaluate OA severity (Fig. 2B). On the medial compartments of the joint, the tibial plateau developed earlier (starting at 2 weeks) and more severe OA compared to femoral condyle (starting at 5 weeks) (Fig. 2B) at the same time-points. Although not statistically significant and much milder, lateral compartments showed a similar trend (Fig. 2B).

### TRAP staining

**Figure 3:**
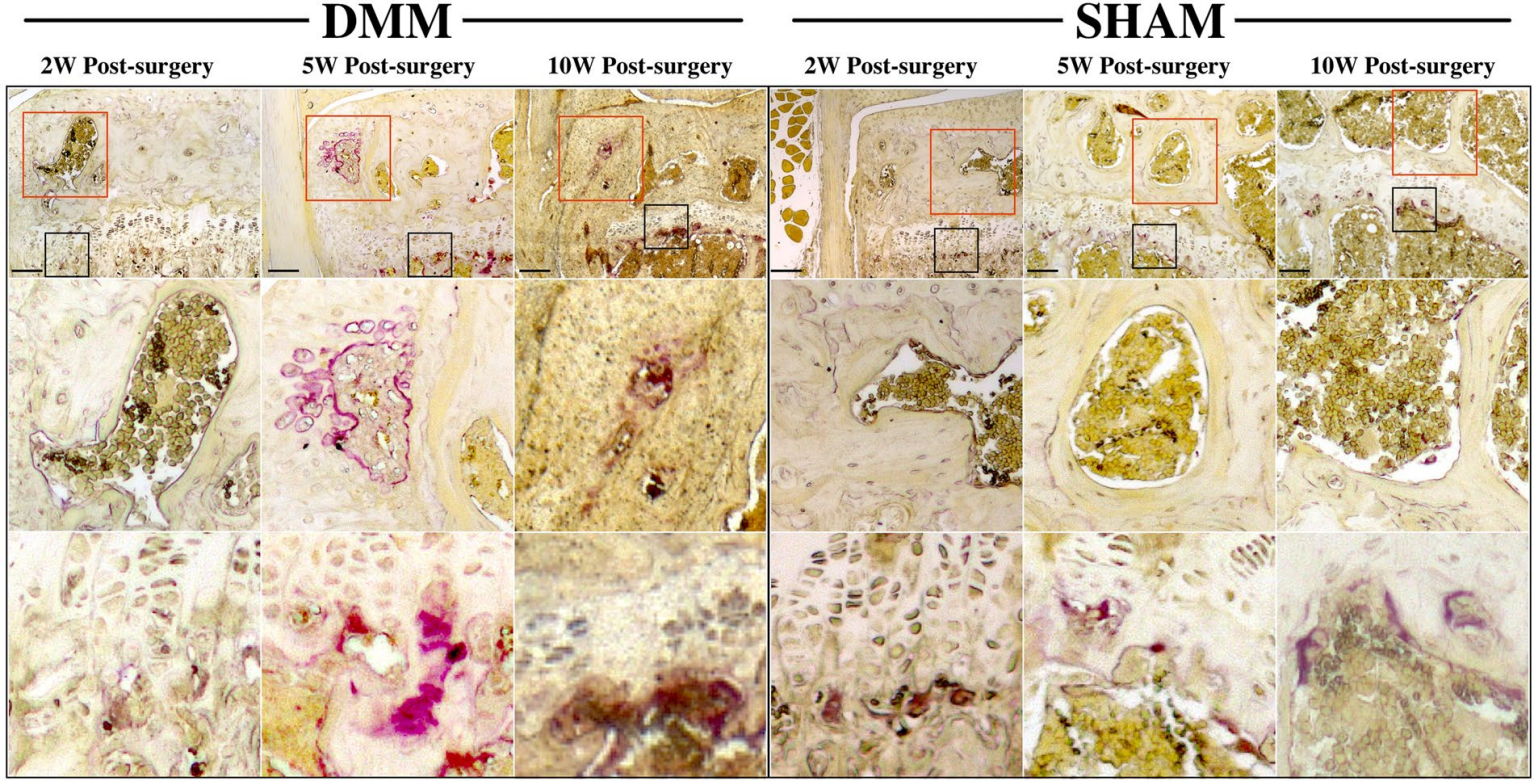
Tartrate-resistant acid phosphatase (TRAP) staining of osteoclast activity after DMM surgery. Osteoclast activity in the subchondral bone appeared increased at 5- and 10-week following DMM surgery, mostly within the osteophyte (red rectangle), but not at 2-weeks. The expected staining at the growth plate was observed (black rectangle). Scale bar: 200 µm.

TRAP positive cells were found in the subchondral bone of DMM mice starting at 5 weeks post-surgery, with most of these cells located at the site of osteophyte formation (Fig. 3). Very little positive staining was found in the subchondral bone of the SHAM group, although the expected staining at growth plate was observed (Fig. 3).

### Subchondral bone changes after DMM surgery

**Figure 4:**
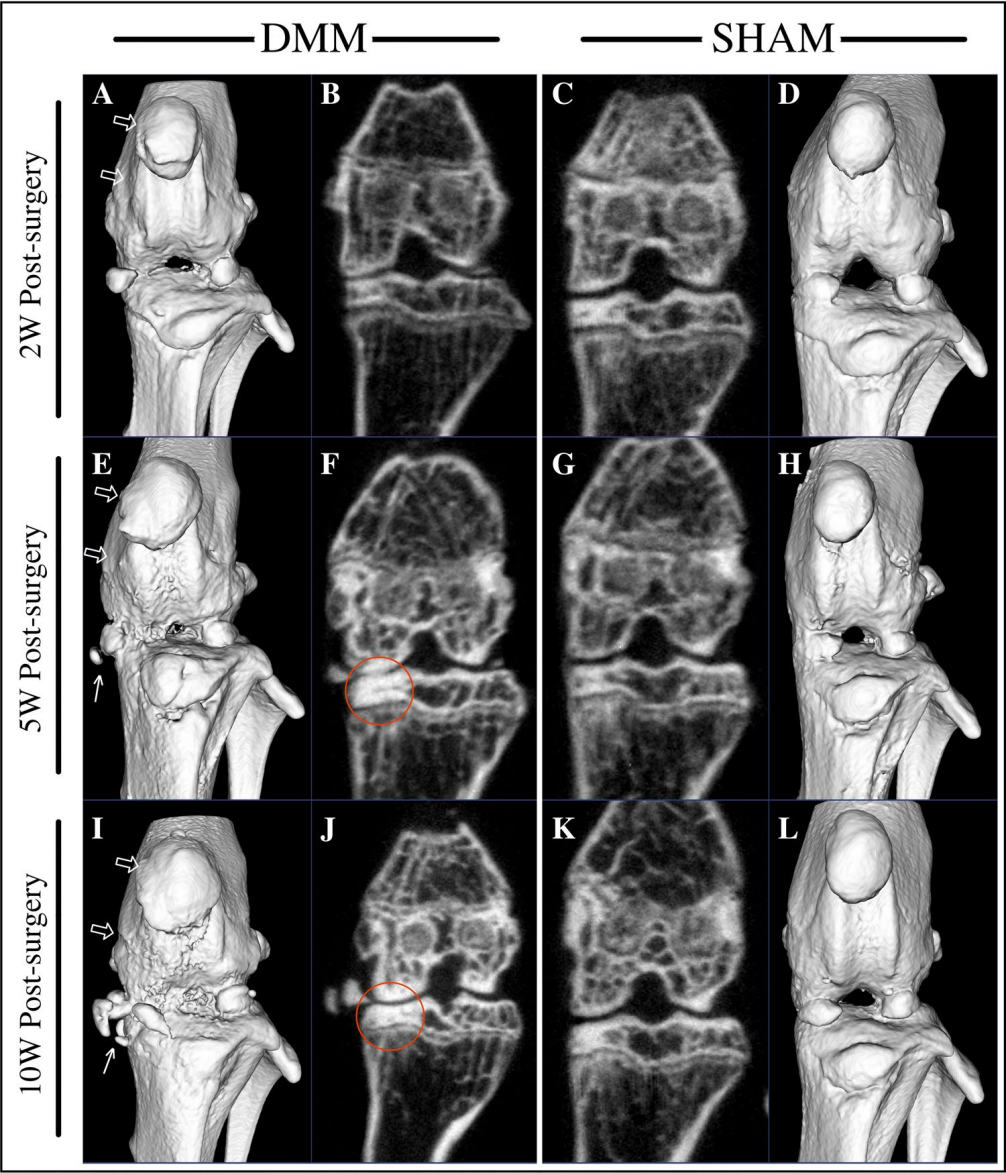
µCT 3D joint reconstruction of DMM-operated mice. Micro-CT analyses demonstrated that joint condition deteriorated through the time course in DMM mice, presenting with uneven bone surface, abnormal patella shape and osteophyte formation (**E**,**I**, arrow) around the joint. A coronal plane that represents the middle of the joint showed bone sclerosis in the medial tibial plateau at 5 and 10 weeks post-surgery in DMM mice. (**F**,**J**, circle).

**Figure 5:**
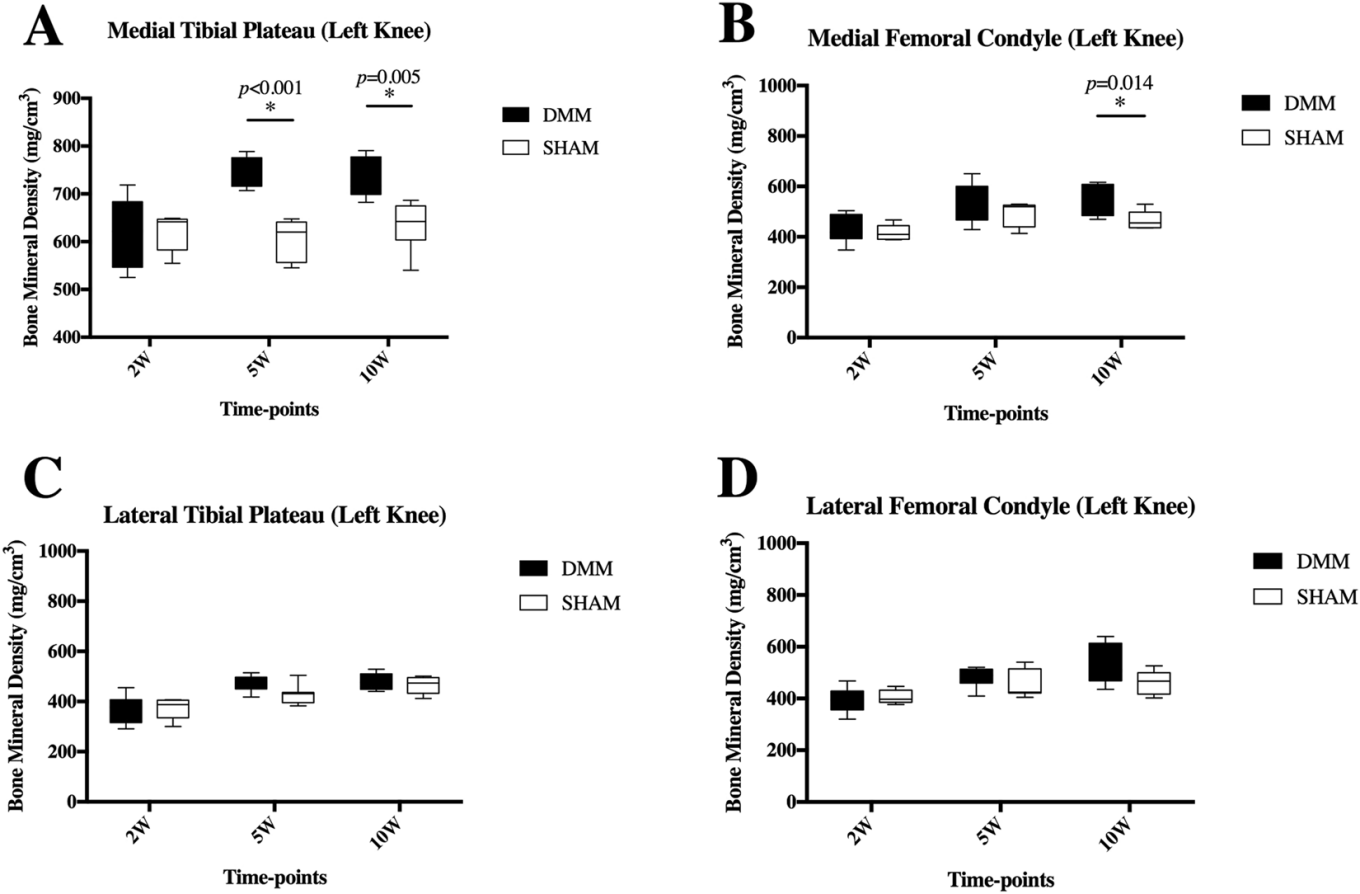
Bone mineral density (BMD) of subchondral bone in the DMM model. At 2 weeks pot-surgery, BMDs of the MTP in DMM mice didn’t showed statistical significant differences comparing to their SHAM controls. However, DMM mice showed a larger spread of BMD at 2 weeks post-surgery (**A**). Clearer differences were detected between DMM and SHAM at both 5 and 10 weeks post-surgery with higher BMDs in DMM mice. (**A**). BMDs of the MFC were higher in DMM mice 10 weeks after surgery (**B**). There was no significant differences found in the lateral compartments between DMM and SHAM at all time-points (**C**,**D**). Error bar stands for the 95% confidence interval.

3D reconstruction of joints from micro-CT analyses is shown in Fig. 4. Irregular bone surface and abnormal patellar shape (osteophyte formations) was noticed in DMM mice as early as 2 weeks post-surgery (Fig. 4A, hollow arrow) and became more obvious at later time-points (Fig. 4E,I, hollow arrow). Osteophyte formation of the MTP was noticed at 5 and 10 weeks post-surgery, with those at 10 weeks being more severe (Fig. 4E,I, white arrow). In the coronal plane in the middle of the knee joint, subchondral bone of the MTP didn’t show difference at 2 weeks in DMM Group compared to SHAM (Fig. 4B), but became denser at later time-points (Fig. 4F,J, circled). BMDs of all compartments are shown in Fig. 5. As seen in the coronal plane images, BMDs of DMM animals showed no statistical differences comparing to their SHAM controls at 2 weeks, with DMM animals displaying a larger spread of their BMDs (Fig. 5A). Clearer differences were detected between DMM and SHAM at both 5 and 10 weeks post-surgery, with increased BMDs in DMM animals at MTP (5 and 10 weeks) and MFC (10 weeks only) (Fig. 5A,B). While the lateral compartments showed no difference between two groups (Fig. 5C,D). No statistical significance was detected among the BMDs of the contralateral right knee (data not shown). BV/TVs were also measured in this study. Statistically differences were only detected in MTP at 2 weeks post-surgery with DMM group presenting a higher BV/TV than their SHAM controls (data not shown). Due to the scan limitation, trabecular thickness or separation couldn’t be calculated in the free version of MicroView 2.5.0 (Parallax Innovations Inc.).

### Subluxation/dislocation of patella after DMM

**Figure 6:**
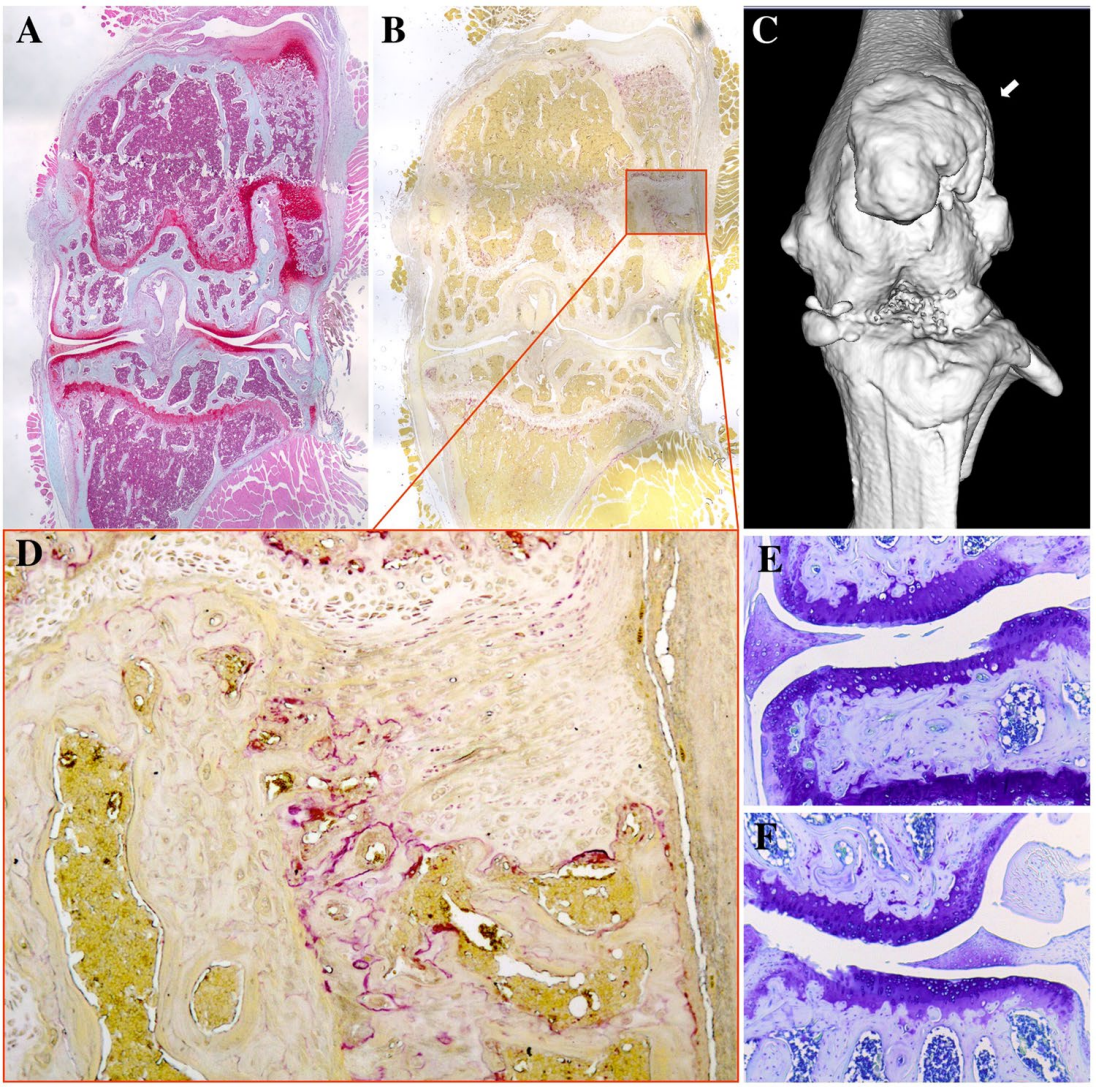
Possible subluxation/dislocation of the patella after DMM surgery. Safranin-O staining showed the out-grown osteophyte stained strongly for GAG (**A**). TRAP staining demonstrated increased number and activity of osteoclasts (**B**,**D**). µCT showed out-grown osteophyte formation (**C**, arrow) and deteriorated joint condition (**C**). Toluidine Blue staining showed cartilage degeneration on medial (**E**) and lateral (**F**) compartments at 10 weeks post-Surgery in DMM mice with patella dislocation.

In a few of our mice (5 of 27, 2 mice of 2-week time-point and 3 of 10-week time-point) that underwent DMM surgery, subluxation/dislocation of the patella was noted as early as 2 weeks post-surgery by micro-CT as well as histological staining (Fig. 6A,C). In these mice, joint condition deteriorated faster as shown by 3D reconstruction and histology. The LTP seemed to have more severe OA than the medial side (Fig. 6E,F), which might be due to abnormal mechanical force on the lateral side of the knee caused by the subluxated/dislocated patella. Osteophyte formation can be observed on the lateral side of the femoral condyle and TRAP staining showed highly increased osteoclast activity within the osteophyte which indicating increased bone remodeling (Fig. 6B,D). Of note, mice with tibia dislocation were not included in this study.

## Discussion

Subchondral bone has been considered as one of the most promising targets for OA drug development^9^. Botter *et al*. reported that in ADAMTS5^−/−^ mice, whose joints were protected from OA showed minor subchondral bone changes after DMM surgery^10^. Carlson and colleagues showed that a thickened subchondral plate was present before apparent cartilage deterioration in a spontaneously developing primate OA model^11,12^. Similar bone changes that occurred earlier than cartilage damage were noticed in other experimental animal models^13^. In Waung’s work, they investigate a digital X-ray microradiography to quantified subchondral BMC in the medial tibial plateau 4 and 8 weeks after DMM surgery and showed increased BMC at both time-points in DMM mice^14^.

This study confirmed in the DMM mouse model, mild cartilage lesion was shown in some of the mice 2 weeks after surgery, presenting with regional proteoglycan loss, chondrocyte clustering and cartilaginous osteophyte formations at the medial side of the surgical joint, which is consistent with the report by Loeser and Das Neves Borges^8,15^. OARSI scores were significantly higher in DMM joints compared with SHAM at all time-points. Mild to moderate articular cartilage damage was observed at 5 and 10 weeks after surgery. Osteophyte formation progressed over time, and endochondral ossification within the osteophyte can be seen at 10 weeks post-surgery.

Gait disparity appeared only at 10 weeks post-surgery but not at earlier time-points, which could be due to the progressive development of OA pain behaviors reported recently^16^. These changes were relatively mild and, surprisingly, affected both hind limbs (not only the operated one). In line with these findings, it has been reported that behavioral changes in rodent OA are usually manifested as reductions in total locomotor activity levels (e.g., active time and distance travelled) other than gait pattern alteration^17^. More detail of gait study in mice could be found in our review^18^.

However, Micro-CT results showed that BMDs of MTP in the DMM-operated animals were more variable than SHAM controls at 2 weeks post-surgery (Fig. 5A), which suggests that the destabilization of medial meniscus possibly leads to early loss of subchondral bone mineralization in the MTP of some mice as early as 2 weeks following DMM surgery. Clearer differences were detected at both 5 and 10 weeks post-surgery, with increased BMD in DMM-operated animals at both the MTP and the MFC (Fig. 5A,B).

It has been reported that a large number of genes were dysregulated as early as 6 hours after DMM surgery by microarray analysis of gene expressed in the whole joint, including known pathogenic OA genes *Mmp3*, *Adamts5*, and *Ccl2*^19^. Furthermore, regulation of selected genes was abrogated after immobilization of the surgical joint, and OA was prevented up to 12 weeks post-surgery by immobilization^19^. This indicated that changes in the mouse joint following DMM were rapid and highly mechanosensitive, despite the fact that surgery itself was considered mild. It appears likely that the subchondral bone responds immediately to the altered mechanical load within the joint, and that this response causes or at least partly contributes to the subsequent pathophysiological events of other structures within the joint. However, almost all genome-wide studies carried out in the DMM model used the whole joint instead of more specific joint tissues due to the small size of the mouse joint. Further investigation of different joint tissues respectively (e.g., subchondral bone, meniscus) would provide additional insights into which tissue responds first.

Low subchondral bone mineralization was present in some animals at the 2-week time-point in our study. However, no statistical significance was found at this time-point. In the meantime, BV/TV of MTP at 2 weeks post-surgery was significantly higher in DMM mice (data not shown), suggesting that increased bone remodeling occurred at very early stages (earlier than 2 weeks post-surgery) of OA in DMM mouse model. In addition, the presence of chondrocyte/osteophyte formation shown in both our and Loeser’s results^15^ as early as 2 weeks post-surgery suggested that changes in the subchondral bone caused by DMM were rapid after the surgery. However, no increased osteoclast activity was found in subchondral bone at the 2-week time-point, which suggests that loss of subchondral bone mineralization due to increased osteoclast function might had occurred earlier.

Mice of 5- and 10- week time-points represent late stages of OA in terms of subchondral bone with bone sclerosis, significantly higher BMD in medial compartments and severe osteophyte formation around the joint compared with the SHAM. Positive TRAP staining can mostly be found in the site of osteophytes at these two later time-points. These data suggest that bone sclerosis occurs in the DMM model similar to human OA^3^ and other animal models.

Another finding of note is the subluxation/dislocation of the patella that occurred in some of the mice following DMM surgery as early as 2 weeks post-surgery (Fig. 6). It altered the alignment of the knee, which complicated the joint destabilization introduced by DMM and interfered with the outcomes. Subluxation/dislocation of the patella imposed an abnormal mechanical load on the lateral femoral condyle, and led to osteophyte formation. Safranin-O staining demonstrated that the out-grown tissue stained strongly for GAGs. Active osteoclasts were detected by TRAP staining, which indicates increased bone remodeling. Articular cartilage degeneration appeared faster as well with this abnormality, especially in the lateral compartments. The reason for this subluxation/dislocation of the patella was unclear. It could be possibly due to a slipped suture of the joint capsule. Our veterinarian who carried out all our DMM surgery, is very experienced based on the large numbers of surgeries he has performed. The DMM model is now the most commonly used OA mouse model, but this complication hasn’t been reported yet to our knowledge. It is possibly one of the reasons that surgical outcomes of DMM vary from lab to lab, and from individual to individual. Most importantly, researchers should be aware of this possible situation caused by the DMM surgery and exclude those animals from the research (as we did in this study).

In summary, subchondral bone changes in the DMM mouse model occurred rapidly, with bone sclerosis occurred as early as 5 weeks post-surgery. This study suggests that subchondral bone changes might occur at the same time as (and possibly earlier than) cartilage changes. Further investigation of early subchondral bone changes driven by osteoclasts/osteoblasts/osteocytes activities as well as gene and protein expressions in the subchondral bone at the early time-points is needed for a better understanding of the molecular mechanism driving bone changes during the initiation of OA in the DMM model.

## Materials and Methods

### Animals and OA induction

All mice were housed on a 12-hour light/dark cycle with unrestricted access to standard mouse food and water. C57/Bl6 wild type mice were randomly designated into 2 groups: DMM and SHAM, and three termination time-points (2, 5 and 10 weeks post-surgery). DMM or sham surgery was performed when mice were 12 weeks of age. All animals were housed and used in accordance with Western University’s Animal Care and Use Guidelines. The protocol was approved by the Animal Care and Veterinary Services of Western University (protocol# 2015-031). Surgery was performed on the left knee joint of mice as previously described^20,21^. A single dose of Amoxicillin (Novopharm, Toronto, Ontario, Canada) was injected subcutaneously right after the surgery to prevent joint infection (20 mg/kg). Buprenorphine (Schering-Plough, Herfordshire, UK) was administered twice (0 and 4 hours after surgery) subcutaneously to minimized pain (0.05 mg/kg). Mice were housed in colony cage after the surgery (up to 6 mice in one cage) and running wheels were put in two days after the surgery to encourage exercise. Excluding the mice that had dislocated patella after surgery (discussed in Results and Discussion Sections), the final size of each group was as followed: DMM group (2W: 6, 5W: 8, 10W: 6), SHAM group (2W: 5, 5W: 7, 10W: 6).

### Gait analysis

Mice were weighed and transferred to the Behavior Study Suite at Robarts Research Institute at Western University two days prior to gait acquisitions. Gait patterns and relative parameters were captured by the Catwalk^®^ gait analysis system (Noldus Inc.) as described^22^. Briefly, mice were placed on a glass platform located in a dark room and allowed to move freely. A light beam from a fluorescent lamp illuminates the platform. Where the paw touches the glass surface, an image of the paw print is recorded by a camera underneath. Gait was captured and parameters was obtained from the Catwalk^®^ software for statistical analysis.

### Limb harvest and µCT

Following gait acquisition, mice were sacrificed by CO_2_ asphyxiation and both hind limbs were dissected immediately and fixed in 4% paraformaldehyde for 24 hours after the removal of skin and extra tissue.

Mouse hind limbs were then scanned using an eXplore RS *in vivo* x-ray µCT scanner (GE Healthcare Biosciences, London, ON, Canada). Calibrating standards for air, water and a cortical bone mimic, SB3 (Gammex RMI), were included with the specimens. The scanning protocol consisted of 900 xy-ray projection images, spaced at an angular increment of 0.4 degrees over a single 6.5-hour gantry rotation at a peak energy of 80 kVp, 450 uA tube current, and 4500 ms per exposure. 5 frames (exposures) per view angle were averaged to reduce noise. Using a filtered-backprojection algorithm, the data sets were reconstructed into 3D volumes with an isotropic voxel spacing of 20 µm, and then linearly rescaled into Hounsfield units (HU) using internal air and water calibration standards. 3D joint morphology reconstruction and BMD measurement were then performed using MicroView software (version 2.1.2). Region of Interest (ROI) for BMD and BV/TV measurement of each compartment was selected as follows in order to cover most part of each compartment: a coronal plane that represented the middle of the joint was first determined. Then on either the medial and lateral regions of the joint, a sagittal plane that represented the center of this region was selected. The ROI was set 0.2 mm under the subchondral bone plate on both tibial plateau and femoral condyle, with the size that cover most part of the subchondral bone in each compartment.

### Histology

Both hind limbs were then put in 5% ethylenediaminetetraacetic acid (EDTA) in phosphate buffered saline (pH 7.0) for decalcification for 10–12 days after the µCT scan. Decalcified limbs were further processed and embedded in paraffin in frontal orientation, with serial sections taken at a thickness of 4 µm and dried in a 37 °C oven for 24 hours, then stored in room temperature. Histology was generally performed as described^23,24^. All sections for staining were deparaffinized in two changes of xylene, rehydrated in ethanol with decreasing concentration ending in water, and stained as follows. General histology of cartilage and bone was assessed in toluidine blue (TB, 0.04% toluidine blue in 0.2 M acetate buffer, pH 3.75–4.25, 5 min), Safranin-O and fast-green (0.02% fast green for 30 minutes, 1% acetic acid for 10 seconds, and 1.5% Safranin O for 3 minutes). Collagen was stained with picrosirius red (PR, 0.1% Sirius red in saturated picric acid solution, 60 min; 0.5% acetic acid washes) and polarized microscope was used to evaluate the size and organization of collagen fibrils of articular cartilage and subchondral bone. Osteoclast activity was detected by tartrate-resistant acid phosphatase (TRAP, Sigma Canada) staining with minor alterations to the manufacturer’s instructions^25^. All images were taken using a Leica DFC295 digital camera mounted on a Leica DM1000 microscope and Leica Application Suite 3.8.0.

### OARSI scoring

OARSI scoring system was used to evaluate OA severity in three sections with different depth after TB staining. Sections were blinded and scored by three different experienced scientists. Average scores were used in statistical analyses^24^.

### Statistical analyses

Statistical analysis was performed in Graph Pad Prism 7. Median and 2.5–97.5 percentile were calculated for OARSI score, BMD (error bar means 95% confidence interval). Gait data (Paw intensity/Weight) followed a lognormal distribution and were log-transformed for parametric test. OARSI scores and BMD data were analyzed by One-way ANOVA. Multiple *t* tests using Holm-Sidak method was then used to analyze the differences between DMM and SHAM at each time-point. In all cases, *p* < 0.05 was considered significant.
